# Women Representation in Surgical Specialties: Reflections about Gender Equity after the 34^th^ Brazilian Surgical Conference

**DOI:** 10.1590/0100-6991e-20223204EDIT01

**Published:** 2022-02-18

**Authors:** SOFIA WAGEMAKER VIANA, LETÍCIA NUNES CAMPOS, MARIA EDUARDA DE FREITAS MESQUITA DO-NASCIMENTO, LÍVIA SOUSA RIBEIRO, VITÓRIA MARQUES DA FONSECA MORAIS, JÚLIA OLIVEIRA DABIEN HADDAD, RODRIGO VAZ FERREIRA, FERNANDA LAGE, JÚLIA LOYOLA FERREIRA

**Affiliations:** 1 - International Student Surgical Network of Brazil - Brasil; 2 - Kursk State Medical University, Faculdade de Medicina - Kursk - Kursk Oblast - Rússia; 3 - Universidade de Pernambuco, Faculdade de Ciências Médicas - Recife - PE - Brasil; 4 - Gender Equity Initiative in Global Surgery - Brasil; 5 - Universidade Federal do Recôncavo da Bahia, Faculdade de Medicina - Santo Antônio de Jesus - BA - Brasil; 6 - Universidade Estadual de Feira de Santana, Faculdade de Medicina - Feira de Santana - BA - Brasil; 7 - Universidade de Itaúna, Faculdade de Medicina - Itaúna - MG - Brasil; 8 - Universidade do Estado do Amazonas, Disciplina de Cirurgia - Manaus - AM - Brasil; 9 - Universidade Federal do Acre, Faculdade de Medicina - Rio Branco - AC - Brasil; 10 - Comissão de Mulheres Cirurgiãs do Colégio Brasileiro de Cirurgiões, TCBC, FACS - Brasil; 11 - Harvey E. Beardmore Division of Pediatric Surgery, The Montreal Children ‘s Hospital, McGill University Health Center - Canada.

**Keywords:** Gender Equity, Health Workforce, Prejudice, Sexism, Equidade de Gênero, Mão de Obra em Saúde, Preconceito, Sexismo

## Abstract

In September 2021, the 34^th^ Brazilian Surgical Conference hosted the “Panel: Women in Surgery” - the only session in the event solely composed of female speakers. Although gender inequities in surgery are well recognized in the international literature, the panel portrayed how distant we are from the desired equity in our country. In addition, the session emphasized the need to broaden the debate and identify the mechanisms for greater inclusion and maintenance of women in the surgical career. In this editorial, we provide a historical overview of gender disparities in the Brazilian surgical ecosystem, highlight the contributing factors to a reduced number of female surgeons, and how the structure of medical societies may influence the rise of women to leadership positions. Accordingly, we discuss the benefits of gender diversity for surgeons, patients, and institutions. Furthermore, we analyze the representation of women in the Brazilian College of Surgeons since its foundation and in the scientific sessions at the conference, demonstrating that more initiatives are required to encourage female representation in the college. Finally, we propose a series of recommendations to foster engagement and contribute to the prosperity of women surgeons in Brazil.

## RESUMO

Em setembro de 2021, o 34º Congresso Brasileiro de Cirurgia sediou o “Painel de debates: Mulheres na Cirurgia” - o único espaço do evento composto somente por mulheres. Embora a existência de iniquidades de gênero na cirurgia seja bem reconhecida na literatura internacional, esse painel retratou como estamos distantes da almejada equidade em nosso país. Além disso, a sessão enfatizou a necessidade de ampliar o debate e identificar os mecanismos para maior inclusão e retenção das mulheres na carreira cirúrgica. Neste editorial, apresentamos panorama histórico e atual das disparidades de gênero no ecossistema cirúrgico brasileiro; destacamos os fatores que contribuem para o número reduzido de cirurgiãs e, como a estrutura das sociedades médicas influencia na ascensão de mulheres para cargos de liderança. Em seguida, discutimos os benefícios da diversidade de gênero para cirurgiões, pacientes e instituições. Ademais, analisamos a representatividade feminina no Colégio Brasileiro de Cirurgiões desde a fundação e nos espaços científicos do congresso, demonstrando que mais medidas serão necessárias para incentivar maior protagonismo feminino no colégio. Finalmente, propomos uma série de recomendações para fomentar o engajamento e contribuir para a prosperidade das cirurgiãs no Brasil.

## EDITORIAL

In September 2021, the 34^th^ Brazilian Congress of Surgery hosted the “Debate Panel: Women in Surgery”, the only space at the event composed only of female guests. The panel was moderated by Dr. Maria Cristina Araujo Maya, professor of general surgery at the State University of Rio de Janeiro, and featured three speakers from different generations: Dr. Elizabeth Gomes dos Santos, general secretary and president of the Commission of Women Surgeons and of the Residency Committee of the Brazilian College of Surgeons (Colégio Brasileiro de Cirurgiões - CBC); Dr. Fernanda Lage Lima Dantas, professor at the Federal University of Acre, member of the Commission of Women Surgeons and full member of the CBC; and Dr. Flavia Yung Ju, plastic surgeon, full member of the CBC and preceptor of the Brazilian Institute of Plastic Surgery. On the panel, the three speakers addressed the low representation of women in the surgical area, the challenges they face, and the factors that contribute to maintaining the status quo. Although the existence of gender inequities in surgery is recognized in the international literature, this panel portrayed how far we are from the desired equity in our country. In addition, it was clear the need to broaden the discussion, identifying means for greater inclusion and retention of women in the surgical careers. Finally, it is still necessary to fight prejudice and to reaffirm the benefits of diversity for surgeons of both genders and for patients, as well as to find ways for medical societies to contribute to the prosperity of female surgeons in Brazil.

Women who choose a surgical career have faced historical challenges for generations. Until 1879, women were not accepted in medical schools in Brazil, a paradigm broken by Dr. Rita Lobato Freitas, the first physician in the country to graduate from the Bahia Faculty of Medicine, in 1887. Gradually, the percentage of women studying medicine increased, reaching 50% of the total number of medical students in 1993, and becoming the majority in 2009^1^. Despite this advance, specialties such as anesthesiology and surgery remain underrepresented by women^1^. Currently, the percentage of women anesthetists is 37.8%, and this percentage is even lower in surgical specialties. While female general surgeons comprise 21.7% of professionals, in neurosurgery, orthopedics, and urology women constitute only 8.8%, 6.5%, and 2.3% of the contingent, respectively^2^. 

Several factors contribute to a reduced number of women opting for a surgical career. Among the reasons mentioned by the conference speakers, two were recognized by a study carried out with Brazilian female doctors as the main challenges: the balance between personal and professional life and the workload^1^. This survey analyzed the responses to a questionnaire applied to 75 non-surgeon female physicians. Approximately 50% of professionals believed that a successful female surgeon should give up aspects of personal life, such as starting a family, and 40% stated that the extensive and inflexible workload makes it difficult to adhere to surgical specialties^1^. Another relevant and undeniable factor for the low representation of women is gender prejudice. Women surgeons are constantly asked about their technical and cognitive skills. However, studies show that male and female residents have the same level of competence in treating their patients^3^ ^,^ ^4^. Regardless of the surgeon’s gender, the postoperative results for patient mortality, complications, readmissions, and length of stay are similar. However, when evaluating the performance of teams, greater female representation is associated with better clinical and surgical outcomes and with greater effectiveness in patient centered communication. Thus, the increase in the number of female surgeons is an effective strategy to expand not only the availability, but the quality of the surgical workforce, in addition to contributing to the increase in the number of procedures in Brazil^5^. 

During the panel, the guests also discussed how the structure of medical societies influences the rise of women to leadership positions^6^. In 2020, the Global Health 50/50 group reviewed the gender practices of 200 international organizations heavily involved in global health policymaking. The report included institutions located in 33 countries and employing 4.5 million individuals. The study showed that 60% of these organizations provide policies to promote gender equity to the public. However, only 30% of executive directors were women. From these percentages, it is estimated that it will take 54 years to achieve gender parity in leadership positions^6^. This reality demonstrates the effects of the phenomenon called “glass ceiling”, which represents the metaphor of an invisible barrier that prevents the promotion of women to higher hierarchical positions, even with equal productivity when compared to their male colleagues^7^. 

 Men and women differ in their experiences and values, which influence both interests and priorities^8^. Consequently, demographic diversity leads to diversity in the intellectual field, scientific production, and health practices of institutions^8^. The evidence is clear in this regard: work groups that are diverse in gender, ethnicity, and socioeconomic context are more productive in generating ideas and innovations, developing more relevant research to the social environment, receiveing more citations, and optimizing decision making^9^ ^,^ ^10^. Thus, organizations that encourage women to be leaders promote a healthy institutional culture that encourages creativity and provides for the well-being of everyone involved^9^. This scenario results in more respectful work environments, with less perpetuation of stereotypes, greater satisfaction, security and corporate stability, and better quality of services offered^8^ ^,^ ^11^. A context open to diversity also allows positive self-perceptions of leadership. The mere presence of women in leadership positions conveys an image of acceptance and recognition of their values. In addition, women in leadership positions recognize and naturally take responsibility for promoting other women in their careers, a principle known as “lifting as you climb”^12^. This principle, associated with a mentoring culture, can have a great impact on the development of the next generations of women surgeons, relieving them from facing the same barriers that their predecessors have endured^8.12^. 

**Figure 1:**
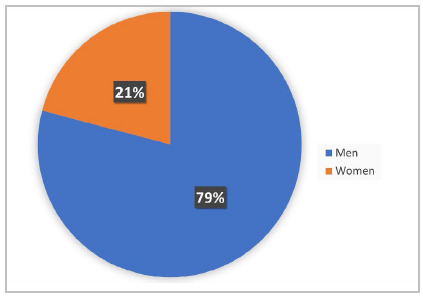
Distribution of guests by gender at the 34 ^th^ Brazilian Congress of Surgery. The graph analyzes the number of guests by gender in the sessions of this edition of the congress. To make this chart, we considered the positions of moderator, debater, and speaker, and all sessions of the congress’ online programming. Of the 455 guests, 95 were women (21%) and 360 were men (79%).

*Source: Online Schedule of the 34* ^*th*^ *Brazilian Congress of Surgery.*

**Figure 2:**
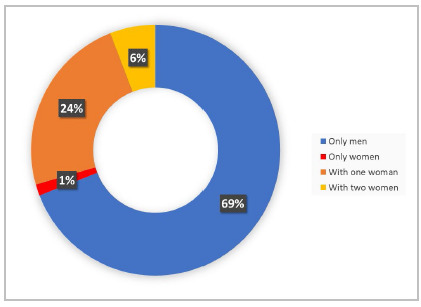
Female representation in the sessions of the 34 ^th^ Brazilian Congress of Surgery. This chart analyzes the distribution of women and men in the 68 sessions of the congress’ scientific program, in the form of panels, round tables, and activities. To make this chart, we considered the positions of moderator, debater, and speaker. Of the 68 sessions, one was composed only of women (1%), 16 had one woman (24%), four had two women (6%), and 47 were composed only of men (69%).

*Source: Online Schedule of the 34* ^*th*^ *Brazilian Congress of Surgery.*

 Although the CBC was founded in 1929, the institution had its first female director in 1961, Dr. Helga da Rocha Pitta, in the position of general secretary. Between 1963 and 1991, no woman held the board of directors of the CBC. This long hiatus was interrupted in 1992, when Dr. Angelita Habr-Gamma became vice president. So far, no woman has held the positions of president, second president, or chairman of the central core of the institution^13^. In 2018, the CBC created the Commission of Women Surgeons, which aims to disseminate ideas, share difficulties, encourage, and suggest actions that can positively transform the lives of female surgeons^14^. However, at the 34^th^ Brazilian Congress of Surgery, only 21% (95/455) of the speakers were women (Figure 1). Furthermore, only 31% (21/68) of the scientific programming spaces contained at least one female moderator, debater, or speaker (Figure 2).

 Under these circumstances, the promotion of gender equity in the surgical workforce encompasses not only a matter of social justice, but also of better working conditions, health policies, and provision of services to patients^3^. As representatives of the International Student Surgical Network of Brazil (InciSioN Brazil) and of the Gender Equity Initiative in Global Surgery Brazil (GEIGS Brazil), we support the building of a diverse and an inclusive medical society in which gender minorities can thrive in their careers. Thus, we propose the following recommendations to encourage female engagement in surgery:

1. Promote education on gender biases (both implicit and explicit) through mandatory institutional courses on diversity, equity, and inclusion for medical students, resident physicians, and practicing physicians, as well as other health professionals;
2. Strengthen institutional notification and response mechanisms in cases of gender discrimination and moral and sexual harassment;
3. Promote equal mentoring and career planning programs with counseling, guidance, and emotional and professional support;
4. Implement family planning initiatives during graduation and residency, providing information on contraceptive methods, fertility, and assisted reproduction, as well as compatible maternity and paternity leave, flexible hours, and day care in workplaces, with adequate facilities for breastfeeding, among others;
5. Promote gender diversity when selecting speakers, moderators, and members of congress organizing committees, to achieve greater equity during the event, including also other genders and ethnic minorities;
6. Develop a diverse and inclusive organizational culture in the context of medical societies in different areas of expertise.

The Brazilian Congress of Surgery is one of the main spaces to promote scientific discoveries, generate debates, and communicate discoveries in the surgical area. Therefore, the event selects which people and ideas are considered relevant to medical-scientific development. In its 34^th^ congress, the CBC was innovative by enabling protagonism to women surgeons and diversity with the “Panel discussion: Women in Surgery”. More measures like these will be needed to allow the Congress and the College to become more inclusive environments. Incorporating diversity and equity as institutional values enable the inclusion of different perspectives and leadership styles that benefit patients, surgeons, and organizations. Thus, medical societies can and should be spokespersons for these changes, as well as agents that promote women’s prosperity in surgery. 
